# TLR7/8 signaling activation enhances the potency of human pluripotent stem cell-derived eosinophils in cancer immunotherapy for solid tumors

**DOI:** 10.1186/s40164-025-00613-y

**Published:** 2025-03-01

**Authors:** Sheng Zhu, Zhengyang Zhou, Ruixin Gu, Zixin Zhao, Yingfeng Zhang, Yudi Miao, Qi Lei, Tianxing Liu, Guokai Wang, Chenyi Dai, Yi Huo, Jinghao You, Lejun Lv, Cheng Li, Ming Yin, Chengyan Wang, Hongkui Deng

**Affiliations:** 1 MOE Key Laboratory of Cell Proliferation and Differentiation, School of Life Sciences, Peking-Tsinghua Center for Life Sciences, Peking University, Beijing, 100871 China; 2 State Key Laboratory of Natural and Biomimetic Drugs, School of Basic Medical Sciences, Peking University Health Science Center, Peking University, Beijing, 100191 China; 3 Center for Bioinformatics, Center for Statistical Science, School of Life Sciences, Peking University, Beijing, 100871 China; 4 Beijing Vitalstar Biotechnology, Beijing, 100012 China

**Keywords:** Eosinophils, Solid tumor, Immunotherapy, Pluripotent stem cells, T-cell infiltration, TLR7/8 signaling, R848, Lung influx, Safety

## Abstract

**Background:**

Efficient tumor T-cell infiltration is crucial for the effectiveness of T-cell-based therapies against solid tumors. Eosinophils play crucial roles in recruiting T cells in solid tumors. Our group has previously generated induced eosinophils (iEOs) from human pluripotent stem cells and exhibited synergistic efficacy with CAR-T cells in solid tumor inhibition. However, administrated eosinophils might influx into inflammatory lungs, posing a potential safety risk. Mitigating the safety concern and enhancing efficacy is a promising development direction for further application of eosinophils.

**Methods:**

We developed a new approach to generate eosinophils with enhanced potency from human chemically reprogrammed induced pluripotent stem cells (hCiPSCs) with the Toll-like receptor (TLR) 7/8 signaling agonist R848.

**Results:**

R848-activated iEOs (R-iEOs) showed significantly decreased influx to the inflamed lungs, indicating a lower risk of causing airway disorders. Furthermore, these R-iEOs had enhanced anti-tumor functions, preferably accumulated at tumor sites, and further increased T-cell infiltration. The combination of R-iEOs and CAR-T cells suppressed tumor growth in mice. Moreover, the chemo-trafficking signaling increased in R-iEOs, which may contribute to the decreased lung influx of R-iEOs and the increased tumor recruitment of T cells.

**Conclusion:**

Our study provides a novel approach to alleviate the potential safety concerns associated with eosinophils while increasing T-cell infiltration in solid tumors. This finding offers a prospective strategy for incorporating eosinophils to improve CAR-T-cell immunotherapy for solid tumors in the future.

**Graphical Abstract:**

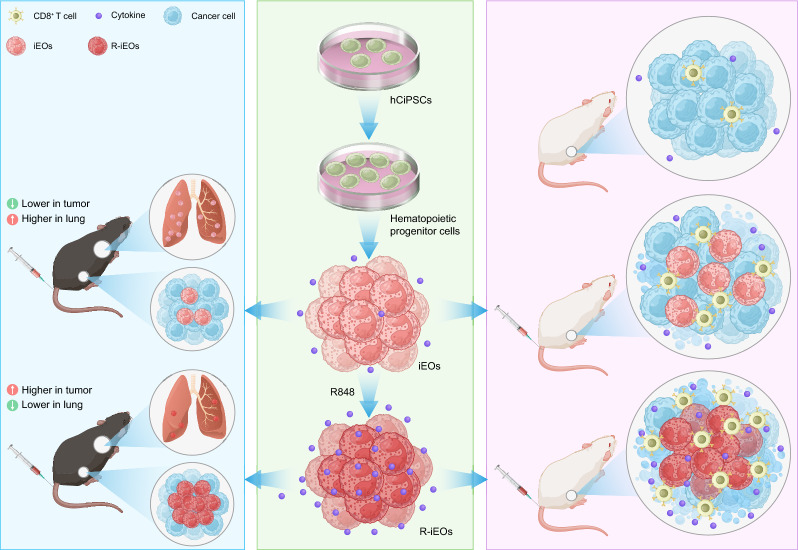

**Supplementary Information:**

The online version contains supplementary material available at 10.1186/s40164-025-00613-y.

## Background

The efficient infiltration of T cells into tumors is pivotal for an effective antitumor immune response in solid tumors [1]. However, developing effective solutions that increase T-cell infiltration into solid tumors to improve the therapeutic efficacy of cancer immunotherapy remains a challenge [2]. Eosinophils, as innate immune cells, play crucial roles in mediating the T immune response in solid tumors and are promising candidate cells for developing novel immune therapies [3, 4]. Eosinophils possess the unique advantage of rapid tumor infiltration early in the immune response [5, 6], as the expression of multiple chemokines and alarmin receptors [7–10] facilitates their migration into solid tumors through signals released from necrotic tumor cells [11, 12]. The infiltrated eosinophils attract T cells and further facilitate T-cell function against tumors by modulating the vasculature and other immune cells [12]. Recently, an increasing number of clinical studies have revealed a positive correlation between a greater number of eosinophils in peripheral or intratumoral regions and a better prognosis [13–15]. Most recently, eosinophils have been shown to play crucial roles in mediating the infiltration of anti-CD19 CAR-T cells during radiotherapy, with the aim of clearing B-cell non-Hodgkin lymphoma (B-NHL) cells [16]. Eosinophils are crucial for the immune checkpoint blockade (ICB) response as they enhance CD8^+^ T-cell function in patients [17]. These above studies have highlighted the important roles of eosinophils in mediating T-cell immune responses, thus positioning them as promising candidates for facilitating T-cell infiltration to combat solid tumors.

However, the limited primary cell resources available for eosinophils have greatly hindered their application. Eosinophils have a short lifespan and are present in limited numbers in the peripheral blood. To resolve this problem, in 2021, we developed a protocol to derive large quantities of induced eosinophils (iEOs) from human pluripotent stem cells (hiPSCs) [18]. These hiPSC-derived iEOs exhibited potent tumor suppression and exhibited enhanced anti-solid tumor effects when combined with CAR-T cells.

Nevertheless, utilizing eosinophils in further applications encounters a potential safety concern. An increase in the number of circulating eosinophils following eosinophil administration may cause an excessive influx of eosinophils into inflammatory lungs, thus posing a potential risk of asthma or other severe airway disorders, including hypereosinophilic syndromes and airway hyperreactivity [19, 20]. Therefore, overcoming the safety concerns by reducing the migration of eosinophils to inflamed lungs is necessary. Eosinophils’ contributions to the progression of respiratory diseases are mainly induced by the Th2-skewed immune response [21]. The expression of chemokines and the inflammatory state of eosinophils are the major factors that regulate their recruitment to the lungs [22]. Modulating the Th2-immune status or inflammatory state may be a promising approach to reduce eosinophil migration to the lungs. Toll-like receptor (TLR) 7/8, which plays an important role in the immune response, is implicated in modulating the expression of cytokines and chemokines [23]. TLR7/8 agonists have been shown to inhibit the skewing of immune responses toward Th2 responses [24]. In experimental asthma models of mouse and nonhuman primates, TLR7/8 agonist treatment alone could reduce eosinophil count in bronchoalveolar lavage fluid and suppress the disease [24, 25]. Thus, we hypothesize that activating TLR7/8 signaling might be an effective approach to potentially modulate eosinophil influx into the lungs to address the safety concerns associated with eosinophils.

In this study, we activated iEOs differentiated from hCiPSCs using the TLR7/8 agonist R848 and examined the effects of R-iEOs on lung chemotaxis in an inflammatory mouse model. We further explored their ability to penetrate solid tumors and recruit T cells. We showed that R-iEOs have reduced influx to inflamed lungs and, surprisingly, exhibited a preference for trafficking to tumor sites. Additionally, they significantly increased T-cell infiltration into tumors. Therefore, our study provides a potential strategy to mitigate the safety concerns associated with eosinophils and to improve T-cell-based therapy for solid tumors.

## Methods

### Mice

Mouse experiments were conducted according to the protocol approved by the Institutional Animal Care and Use Committee (IACUC) of Peking University. All NOD.Cg-*Prkdc*^*scid*^* Il2rg*^*tm1vst*^/Vst (NPG) mice (stock number: vs.-AM-001) were purchased from Beijing Vitalstar Biotechnology. In-house bred C57BL/6J mice and founder mice were purchased from Jackson Laboratory. The mice ranged from 5 to 10 weeks of age.

### Cell culture

The hCiPS cell line was established in our laboratory. hCiPSCs were cultured in Matrigel (BD Biosciences, Franklin Lakes, NJ; Cat: 354230)-coated plates with mTeSR Plus medium (STEMCELL Technologies, Vancouver, Canada; Cat: 100–0276) under 20% O_2_ and 5% CO_2_ at 37 °C. hCiPSCs were passaged by treatment with ReLeSR (STEMCELL Technologies; Cat: 05873) for 5–6 min at 37 °C, and the cells were collected and split at 1 to 6—1 to 10 ratios with mTeSR Plus medium containing 10 μM Y-27632 (Selleck, Huston, TX; Cat: S1049).

HCT116-Luc cell line was constructed in our previous work with the original HCT116 cell line, which was obtained from the National Infrastructure of Cell Line Resource (Beijing, China). The cell line was cultured in Dulbecco’s modified Eagle medium (DMEM; Gibco, New York, NY; Cat: C11965500BT) supplemented with 10% fetal bovine serum (FBS; GeminiBio, West Sacramento, CA; Cat: 124 900–108) and 1% penicillin/streptomycin (PS; Gibco; Cat: 15140122) under 20% O_2_ and 5% CO_2_ at 37 °C. Trypsin–EDTA (0.25%) (Gibco; Cat: 25200056) was used for passaging.

### Eosinophil differentiation from hCiPSCs

hCiPSCs were cultured in Costar^®^ 6-well Clear Flat Bottom Ultra Low Attachment plates (Corning, Franklin Lakes, NJ; Cat: 3471) at a density of 6 × 10^5^ per well in mTeSR Plus medium containing 50 nM Chroman 1 (MCE, Shanghai, China; Cat: HY-15392) and 5 μM Emricasan (MCE; Cat: HY-10396) 1 day before differentiation. On differentiation day 0, 20 ng/mL human basic fibroblast growth factor (bFGF; Origene, Rockville, MD; Cat: TP750002) and 2 μM Y-27632 and CHIR-99021 (10 μM) were added to early induction medium supplemented with RPMI 1640 (Gibco; Cat: 22400089), 2% B27 (without vitamin A; Gibco; Cat: 12587010), 1% nonessential amino acids (NEAA; Gibco; Cat: 11140050), 1% GlutaMAX (Gibco; Cat: 35050061), 1% PS, 50 μg/mL L-ascorbic acid 2-phosphate sesquimagnesium salt hydrate (ascorbic acid; Sigma-Aldrich, St. Louis, MO; Cat: A8960), and 451 μM α-thioglycerol (Sigma-Aldrich; Cat: M6145). From day 2 to day 6, early induction medium containing 5 ng/mL BMP4 (StemImmune LLC, Livermore, CA; Cat: HST-B4-0100), 50 ng/mL human vascular endothelial growth factor (VEGF; StemImmune LLC; Cat: HVG-VF5-1000), and 50 ng/mL bFGF and 10 μM SB-431542 (Selleck; Cat: S1067) were added. From day 7 to day 12, 50 ng/mL recombinant human stem cell factor (SCF; StemImmune LLC; Cat: HHM-SF-1000), 50 ng/mL recombinant human thrombopoietin (TPO; StemImmune LLC; Cat: HHM-TP-1000), 50 ng/mL recombinant human FMS Like Tyrosine Kinase 3 Ligand (FLT3L; StemImmune LLC; Cat: HHM-FT-1000), and 50 ng/mL human recombinant interleukin-3 (IL3; StemImmune LLC; Cat: HCT-I3-1000) were supplemented in hematopoietic induction medium, which consisted of IMDM (IMDM; Gibco; Cat: C12440500BT) containing 2% B27 (without vitamin A), 1% GlutaMax, 1% NAC, 1% PS, 50 μg/mL ascorbic acid, 451 μM α-thioglycerol, 30 μM NAC (Sigma; Cat: A7250-5G) and 2 μM minocycline hydrochloride (Selleck; Cat: 3268). After day 12, hematopoietic induction medium supplemented with 20 ng/mL IL3 and 20 ng/mL human recombinant interleukin-5 (IL5; Novoprotein, Shanghai, China; Cat: CI59) was used to induce eosinophil maturation. During maturation, half of the medium was changed every 2 days; in particular, the culture plate was set uninterrupted for 5 min to allow cell settlement, 2 mL of medium was carefully removed from the supernatant without disrupting the bottom cell sediment, and 2 mL of fresh medium was then added.

### R848 activation of hCiPSC-derived eosinophils

R848 was purchased from MCE (Cat: HY-13740). hCiPSCs-derived eosinophils (D26-D36 post differentiation) were cultured with hematopoietic induction medium supplemented with 20 ng/mL IL3, 20 ng/mL IL5, and 1 µg/mL R848 for 24 h before analysis and experiments.

In inhibition experiment, inhibitors (detailed information in Supplementary Table 2) were added to hematopoietic induction medium supplemented with 20 ng/mL IL3 and 20 ng/mL IL5 at indicated concentration. 5 × 10^5^ hCiPSCs-derived eosinophils (D26-D36 post differentiation) were cultured in 1 mL of indicated inhibitor containing medium for 5 h, 1 µg/mL R848 was then supplemented into the medium. After a prolonged culture for 24 h, cells were harvested for downstream analysis.

### Flow cytometry analysis

For surface marker detection, cultured cells were collected at the indicated times, digested with Accutase (Millipore, Molsheim, France; Cat: SCR005) at 37 °C for 5 min, diluted with an equal volume of PBS (Sangon, Shanghai, China; Cat: E607008-0500), centrifuged at 1,800 rpm for 3 min to obtain cell pellets, and resuspended in PBS containing 0.5% bovine serum albumin (BSA; Sigma-Aldrich, Cat: A1470–100G) and 5% True-Stain Monocyte Blocker (Biolegend, San Diego, CA; Cat: 426103) and 5% Human TruStain FcX (Biolegend; Cat: 422302)to form a single-cell suspension. After 15 min incubation in room temperature, the indicated antibodies were added, and the cells were incubated for 15 min in the dark at room temperature. Then, the cells were washed three times with PBS, suspended in 200 µL of PBS, and filtered through a 40 μm nylon cell strainer for analysis. Each antibody (0.2 µL) was added to each sample. The antibodies used were as follows: 7-AAD (BD; 559925), APC anti-human CD45 (Biolegend; Cat: 304021), PE anti-human CD69 (Biolegend; Cat: 310906), PE anti-human CD11b (Biolegend; Cat: 301306), and PE-Cy7 anti-human SIGLEC-8 (Biolegend; Cat: 347112). The isotype control antibodies used were as follows: Brilliant Violet 421™ Mouse IgG1, κ Isotype Ctrl Antibody (Biolegend; Cat: 400157), PE/Cyanine7 Mouse IgG1, κ Isotype Ctrl Antibody (Biolegend; Cat: 400125), and PE Mouse IgG1, κ Isotype Ctrl Antibody (Biolegend; Cat: 400111). For intracellular staining of EPX, cultured cells were collected at the indicated times and digested into single-cell suspensions as described above. Then, the cells were stained with Fixable Viability Stain 575 V (BD; Cat: 565694) according to the manufacturer’s instructions. Next, the cells were fixed and permeabilized using a BD Cytofix/Cytoperm^™^ Fixation/Permeabilization kit (BD; Cat: 554714), and then they were stained with an anti-EPX antibody (Abcam, Hangzhou, China; Cat: ab190715) according to the manufacturer’s instructions; a proportion of the cells were stained with Purified Mouse IgG1, κ Isotype Ctrl Antibody (Biolegend; Cat: 400165) as an isotype control. The cells were then washed twice with 1 × BD Perm/Wash buffer before being incubated with Alexa Fluor 488-AffiniPure donkey anti-mouse IgG (1:200 dilution; Jackson ImmunoResearch, Philadelphia, PA; Cat: 715-545-150) at 37 °C for 15 min. Then, the cells were washed twice and filtered through a 40 μm nylon cell strainer for analysis.

To analyze the infiltration of hCiPSC-derived eosinophils into solid tumors and organs, mice were euthanized, and tumors and organs were isolated. Isolated tumors and organs were cut into 1 mm pieces with scissors and then digested by incubation at 37 °C with 1 µg/mL collagenase IV (Sigma; Cat: 17104019), 1 µg/mL collagenase II (Sigma; Cat: C2-28-100MG) and 1 mg/mL DNAse I (Sigma; Cat: DN25-1G) for 30 min. Single cells were pipetted into a tube after digestion and collected by centrifugation at 1,800 rpm for 5 min. The cells were washed three times with PBS, and filtered through a 40 μm nylon cell strainer for flow cytometry. Flow cytometry analysis was conducted using Cytoflex S (Beckman Coulter, Brea, CA).

### Electron microscopy

Eosinophils and R848-treated eosinophils were fixed with 2% paraformaldehyde/2.5% glutaraldehyde in 0.1 M phosphate buffer (pH 7.4) for 5 min at 37 ℃, and then incubated for another 30 min at room temperature and overnight at 4 ℃. After being rinsed several times in phosphate buffer, the cells were postfixed in 2% OsO_4_ supplemented with 1.5% potassium ferrocyanide for 2 h at room temperature. Following several washes in distilled water, the samples were stained with 2% aqueous uranyl acetate overnight at 4 °C. After washing several times in distilled water, the cultures were dehydrated in a graded alcohol series and subsequently embedded in Spurr’s resin (SPI supplies, West Chester, PA). Ultrathin Sects. (70 nm) were cut with a diamond knife on an ultramicrotome (UC7, Leica Microsystem, Wetzlar, Germany) and collected on copper grids with a single slot. Sections were stained with uranyl acetate and lead citrate and observed under an electron microscope (Tecnai G2 Spirit; FEI, Hillsboro, OR) at 120 kV.

### RNA-seq and bioinformatics analysis

Total RNA was isolated from eosinophils treated with or without R848 using a Direct-zol^™^ RNA MiniPrep kit (Zymo Research, Orange, CA; Cat: R2051). RNA-seq libraries were constructed using the NEBNext^®^ Ultra^™^ II RNA Library Prep Kit for Illumina^®^ (NEB, Rowley, MA; #E7775L). The fragmented paired-end libraries were sequenced using an Illumina NovaSeq 6000. All sequencing was performed at Novogene. For bioinformatics analysis of the RNA-seq data, we used trim-galore 0.6.2 (https://www.bioinformatics.babraham.ac.uk/projects/trim_galore/) to remove detected adapters from fastq files with the parameter “-q 20 –phred33 –stringency 1 –length 20” and performed quality control using FastQC 0.11.8 (https://www.bioinformatics.babraham.ac.uk/projects/fastqc/). Next, we aligned these reads to the hg19 reference genome using STAR 2.7.10a (https://github.com/alexdobin/STAR) and sorted output bam files using samtools 1.13 (https://github.com/samtools/). Then we calculated count gene expression in htseq-count 0.11.3 (https://github.com/genepattern/HTSeq.Count). Finally, we used R language to build a count matrix for downstream analysis. The gene count matrix was normalized for visualization using R package DESeq2 1.38.3 (https://github.com/thelovelab/DESeq2).

### Quantitative real-time PCR

Total RNA was isolated from the indicated cells with a Direct-zol^™^ RNA MiniPrep kit according to the manufacturer’s protocol. A TransScript^®^ One-Step gDNA Removal and cDNA Synthesis SuperMix (Transgen, Beijing, China; Cat: AT311) kit was used to synthesize cDNA from total RNA. Quantitative real-time PCR was performed in triplicate from at least three biological samples with a Bio-Rad CFX Connect^™^ Real-Time PCR Detection System (Bio-Rad, Hercules, CA; Cat: 1855201). Quantitative PCR was carried out in a volume of 20 µL using Taq Pro Universal SYBR qPCR Master Mix (Vazyme; Cat: Q712). The PCR protocol was performed as follows: first, 95 °C for 3 min to activate the polymerase and pre-denaturation of the template, followed by 40 cycles at 95 °C for 10 s (for denaturation) and 60 °C for 30 s (for annealing and extension). The values for mRNA expression were normalized to those of untreated eosinophils. The primer sets used to detect single genes are listed in Supplementary Table 1.

### Isolation of human primary T cells from cord blood

All cord blood mononuclear cells used in our study were obtained from healthy donors who provided informed consent (Blood Center of Beijing Red Cross Society). Cord blood was first diluted with sterile PBS supplemented with 2% PS, aliquoted gently into human lymphocyte separation medium (DRKEWE, Shenzhen, China; Cat: DKW-KLSH-0100), and centrifuged at 1,500 rpm for 20 min according to the manufacturer’s instructions to obtain blood cells separated into different layers. The buffy coat in the middle layer, which contained mainly mononuclear cells, was collected and diluted with sterile PBS, centrifuged at 1,500 rpm for 20 min, washed again with PBS, and centrifuged at 1,500 rpm for 5 min. The cell pellets were then collected and stained with CD8 MicroBeads (Miltenyi, Bergisch Gladbach, Germany; Cat: 130–045-201) in PBS containing 0.5% BSA for 15 min in the dark at room temperature. Next, cells were washed with PBS containing 0.5% BSA, and the CD8^+^ T cells were sorted using a magnetic-activated cell sorting (MACS) system.

### Activation and CAR transduction of human T cells

Human T lymphocytes were cultured in ImmunoCult-XF T-Cell Exp Medium (STEMCELL Technologies; Cat: 10981) supplemented with 10% FBS and 20 ng/mL recombinant human IL-2 (IL2; Novoprotein; Cat: C013). After 48 h of activation with ImmunoCult™ Human CD3/CD28/CD2 T-Cell Activator (25 µL/mL) (STEMCELL Technologies; Cat:10970), 8 μg/mL polybrene (Millipore; Cat: TR-1003-G) and 20 ng/mL IL2 were added to each well, and T cells were transduced twice over the next 48 h with HER2-CAR lentivirus by spinoculation for 1 h. The transduction efficiency was determined by flow cytometry.

### Transwell assay

Transwell assays were conducted with 24-well cell culture inserts (6.5 mm diameter, 5 µm pore, PC membrane) (Labselect, Wujiang, China; Cat: 14331). The assay was used to determine the degree of eosinophil chemoattraction of T cells. The upper chamber of the insert contained 1 × 10^5^ primary T cells at a volume of 200  µL. The bottom chamber contained 400 µL of coculture supernatant of HCT116 tumor cells alone or with R848-treated/untreated eosinophils, or of fresh culture medium or supernatant from medium cultured with eosinophils or R848-treated eosinophils for 24 h. After 6 h of incubation, cells in the bottom chamber were counted. For antibody blockade, Human CXCL10 Antibody (R&D systems, Minneapolis, MN; Cat: MAB266-SP) was added to the bottom chamber at a final concentration of 5 µg/mL. For inhibitors against CXCR3, 1 µM AMG 487 (TargetMol, Shanghai, China; Cat: T10297L) or 1 µM rac-NBI-74330 (TargetMol; Cat: T26035) was used in both the upper and bottom chamber. T cells were incubated in 1 µM AMG 487 or rac-NBI-74330 for 1 h prior to transwell assay.

The assay was used to determine the degree of eosinophil chemoattraction of T cells in the tumoral environment. The upper chamber of the insert contained 1 × 10^5^ primary T cells at a volume of 300 µL. The bottom chamber contained 4 × 10^4^ HCT116-Luc tumor cells alone or with R848-treated/untreated eosinophils. After 6 h of incubation, all cells remaining in the upper chamber were counted.

### Luminex assay of cytokine and chemokine production

HCT116 tumor cell spheroids were incubated with 5 × 10^5^ R-iEOs or untreated eosinophils cocultured with 1 × 10^5^ CD8^+^ T cells. After 24 h of incubation, the supernatant of the culture medium was collected and sent for a Luminex assay at LabEx (Shanghai, China).

### In vivo tumor assay

To evaluate the combined effects of CAR-T cells and R-iEOs, we used CAR-T cells or R-iEOs alone as controls. Tumor cells were injected subcutaneously at a dose of 5 × 10^4^ per mouse on day -3. Three intravenous injections of iEOs or R-iEOs were injected on day 0, day 4, and day 7 at a dose of 5 × 10^6^ for each mouse. Sorted CAR-T cells were injected intravenously one day after eosinophil injection; injections were administered on day 1, day 5, and day 8, at a dose of 1.5 × 10^5^ cells/injection. The control groups were injected with only an equal volume of culture medium to enable comparison with the experimental groups. The tumor burden of each mouse was monitored by in vivo bioluminescent imaging using Xenogen IVIS (Caliper Life Sciences, Boston, MA). Mice were injected intraperitoneally with 150 mg/kg D-luciferin potassium salt (BioMol, Hamburg, Germany; Cat: BM8056), anesthetized with 2,2,2-tribromoethanol (TBE; Sigma‒Aldrich; Cat: T48402), and imaged 5 min later.

### In vivo cell trafficking assay

NSG mice were first injected with 5 × 10^4^ HCT116-Luc tumor cells per mouse. Before injection, T cells or R848-treated/untreated eosinophils were labeled with 1,1′-dioctadecyl-3,3,3′,3′-tetramethylindotricarbocyanine iodide (DiR; MCE, HY-D1048) according to the manufacturer’s instructions. Briefly, the cells were counted, resuspended in medium containing 2.5 µM DiR, and incubated at 37 °C in the dark. Then, the cells were washed with sterile PBS and resuspended in medium at 5 × 10^4^/µL, and 100 µL of labeled cells were injected intravenously into each mouse 6 weeks after tumor injection. At the indicated time points after cell injection, the mice were anesthetized with TBE and imaged via a Xenogen IVIS system with 740 nm excitation and 790 nm emission.

For eosinophil trafficking in the allergic animal model, 0.018 g of Albumin from chicken egg white (OVA; YUANYE, Shanghai, China; S12017-25 g) was dissolved in PBS at a final volume of 6 mL, forming a 3 mg/mL stock solution. On the day of usage, 70 µL of stock solution was diluted 30 times with PBS to 0.1 mg with a total volume of 2.1 mL, after which 2.1 mL of aluminum hydroxide adjuvant (Bioss, Beijing, China; C07-01013) was added to the final mixture. On day 0 and day 7, C57BL/6 J mice were intraperitoneally injected with 200 µL of the OVA mixture. A total of 1 × 10^6^ HCT116-Luc tumor cells were injected subcutaneously on day 8. On days 14 and 15, the mice were challenged with 50 µL of the OVA mixture by intranasal administration. On day 15 after OVA challenge, 5 × 10^6^ DiR-labeled untreated or R848-treated eosinophils were intravenously administered. On day 17, the mice were euthanized, and the lungs and tumors were excised and imaged via a Xenogen IVIS system with 740 nm excitation and 790 nm emission.

### Confocal microscopy

For confocal microscopy, tumors were collected from mice, fixed in 4% paraformaldehyde for 24 h at room temperature, and hyalinized using a BeyoCUBIC Animal Tissue Optical Clearing Kit (Beyotime, Shanghai, China; P0112M) according to the manufacturer`s protocol. Samples were imaged using Fusion 1.1.0.1 software on a BC43 spinning disk confocal microscope (Oxford Instruments, Abingdon, UK) using green (488 nm) and red (638 nm) excitation wavelengths. Z-stack imaging was performed.

### Statistical analysis

Statistical analysis was performed with GraphPad Prism software. The data are shown as individual measurements with the mean and standard deviation or the mean and standard error of the mean. Comparisons between groups were assessed using Student’s T test or one-way ANOVA as indicated. For all analyses, *p* < 0.05 was considered to indicate statistical significance. The statistical significance and n values are described in the figure legends. All the flow analysis data were processed with FlowJo v.10 software.

## Results

### Generation of R-iEOs from chemically reprogrammed induced pluripotent human stem cells

**Fig. 1:**
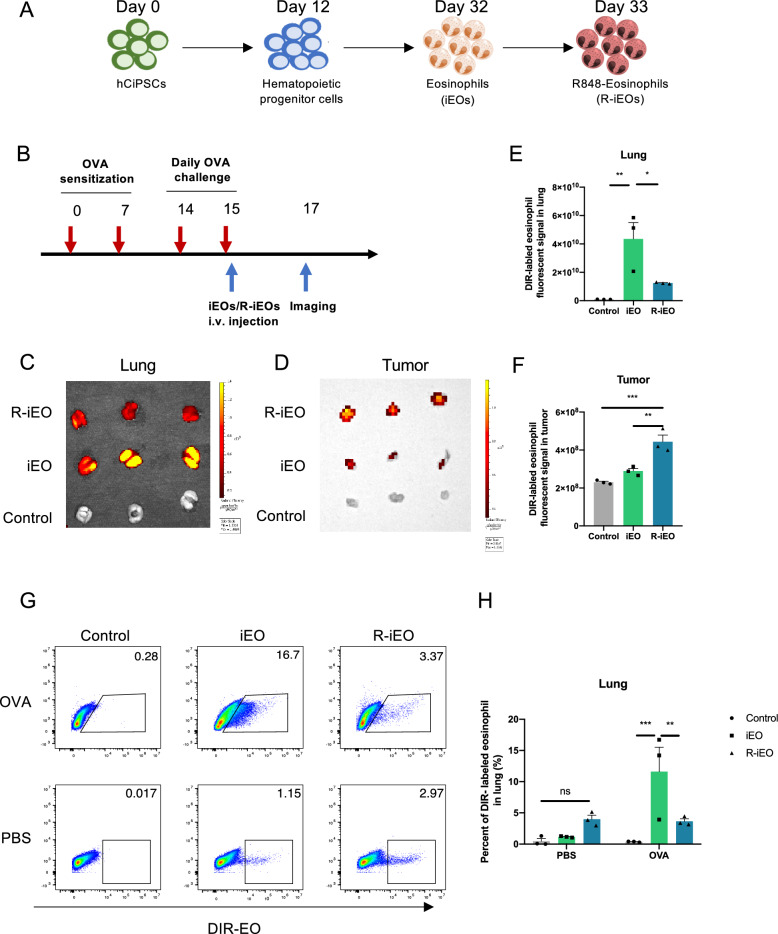
R-iEOs show reduced accumulation in the lungs in an allergic airway inflammation mouse mode. **A** Schematic diagram of the workflow on the induction of R-iEOs. **B** Schematic diagram on the workflow on OVA-induced allergic airway inflammation mouse model. **C** Mice that received R-iEOs had a significantly lower DIR signal in the lung compared with that in iEOs group. (N = 3) **D** Higher R-iEOs accumulation in tumors of OVA-challenged C57BL/6 mice compared with normal iEOs. (N = 3) **E**, **F** Quantification of DIR fluorescence of R-iEOs or normal iEOs accumulation in lung or tumor. (N = 3) (**G**) Flow cytometry analysis and quantification (**H**) of R-iEOs or normal iEOs accumulation in OVA-induced allergic airway inflammation mouse model. The gating strategy is shown in Fig S2A. (N = 3) One-way ANOVA, *p* value * < 0.05, ** < 0.01, and *** < 0.001

Based on our previous study [18, 26, 27], we generated iEOs from hCiPSCs through a stepwise protocol by sequential early mesoderm cell commitment, hemogenic endothelial cell specification, hematopoietic progenitor induction, and eosinophil differentiation. We efficiently generated iEOs from hCiPSCs with nearly 95% EPX expression. hCiPSCs-derived iEOs express multiple TLRs and TLR8 is among the highest (Fig S5A). To generate TLR7/8-modulated iEOs, we treated mature iEO cells with the TLR7/8 agonist R848 for 24 h (Fig. 1A). A flow cytometry panel of eosinophil markers including antibodies against CD45, CD11b, SIGLEC-8, and EPX was used. As shown in Fig S1A-D, R848 activation did not alter the expression of eosinophil markers, including CD45, SIGLEC-8, or EPX, however, CD11b was increased.

### R-iEOs showed reduced accumulation in the lungs in an allergic airway inflammation mouse model

Despite increasing evidence supporting the potential role of eosinophils in cancer immunology, eosinophils might still be associated with lung inflammation, especially when the body is exposed to allergic stimulation in the airway. Thus, the key challenge with eosinophil administration is the risk of high eosinophil accumulation in the airway, which can lead to subsequent eosinophilic inflammation.

To study the hypothesis of whether TLR7/8 signaling modulation on iEOs could play effective roles in reducing their accumulation in the lungs, we established an allergic airway inflammation mouse model via OVA challenge. Then, these allergic mice were injected intravenously with 1,1′-dioctadecyl-3,3,3′,3′-tetramethyl indotricarbocyanine Iodide (DiR)-labeled R-iEOs or normal iEOs. Lungs and tumors were collected and analyzed 2 days after the injection (Fig. 1B). OVA-challenged mice that received normal iEOs injection had more DiR signaling in the lungs compared to PBS-treated mice receiving normal iEOs injection, indicating that these mice experienced an influx of eosinophils in the lungs (Fig. 1C, E). We also observed that some mice experienced airway syndromes, such as sneezing or panting (data not shown). Meanwhile, in OVA-challenged mice, those receiving R-iEOs had a 71.5% reduction in the DiR signals in the lungs compared to those receiving normal iEOs. Surprisingly, we observed greater R-iEO accumulation in the tumors of OVA-challenged mice than in those of normal iEOs-treated mice (Fig. 1D, F). The flow cytometry data confirmed these results (Fig. 1G, H). These data indicated that R-iEOs possess reduced recruitment to the lungs and increased accumulation in tumor sites in mice with allergic airway inflammation.

### R-iEOs infiltration is promoted in the TME

**Fig. 2:**
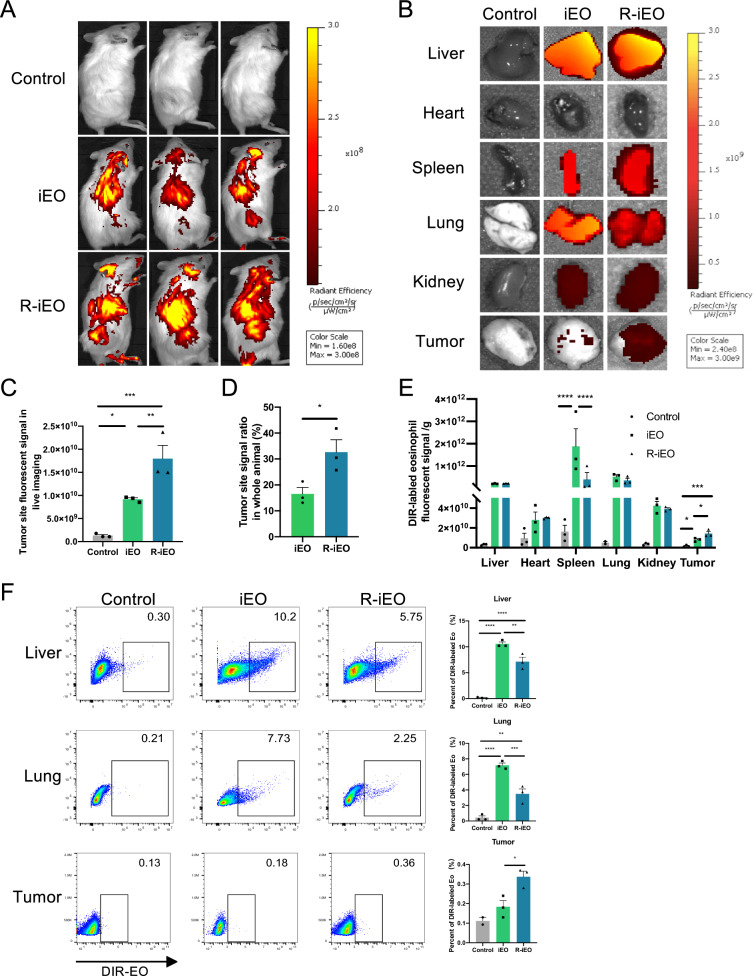
Tumor trafficking and biodistribution of DIR-labeled eosinophils in HCT116 tumor mice models. **A** Preliminary assessment of in vivo DiR-labeled eosinophils tracking using whole animal fluorescence imaging. (N = 3). **B** Representative near-infrared images of different organs and tumors showing biodistribution of DIR-labeled eosinophils at 24 h. (N = 3). **C**, **D** ROI analysis was performed on the tumor site and the whole animal. (N = 3). **E** Quantitative analysis of fluorescence intensity for ex vivo imaging of organs and tumors. (N = 3) **F** Flow cytometry analysis of R-iEOs trafficking in liver, lung, and tumor in mice. The gating strategy is shown in Fig S2B. (N = 3) One-way ANOVA, *p* value * < 0.05, ** < 0.01, *** < 0.001, and **** < 0.0001

To evaluate the infiltration and biodistribution of R-iEOs post-transfer, DiR-labeled eosinophils were injected intravenously into HCT116 tumor-bearing mice. Twenty-four hours after the injection of eosinophils, live imaging, and ex vivo detection using an in vivo imaging system revealed that significantly more DiR signals were detected at the tumor site in mice receiving R848-activated eosinophils than in mice receiving normal iEOs (Fig. 2A–D).

To investigate the biodistribution of eosinophils, an array of mouse organs was collected, weighed, and used to quantitatively analyze the distribution of eosinophils. DiR-labeled eosinophils were present in all tissues examined, including liver, heart, spleen, lung, kidney, and tumor tissues (Fig. 2B). The liver was the primary tissue where eosinophils accumulated 24 h after intravenous administration, followed by the lung, spleen, and tumor (Fig S3A). Taking the total organ weights into account, the fluorescent signal was mainly observed in the spleen, liver and lungs (Fig. 2E). There was a lower signal for R-iEOs in the spleen than for normal iEOs. Importantly, R-iEOs showed greater accumulation at the tumor site than did the nonactivated controls (Fig. 2E, Fig S3B).

We further dissociated tissues and performed flow cytometry to examine the accumulation of DiR-labeled eosinophils in organs. The flow cytometry data showed that R848-activated eosinophils had greater accumulation in tumors and a lower accumulation in the livers and lungs than nonactivated eosinophils (Fig. 2F). These data indicate that R-iEOs have more potential to be recruited to tumor sites following transfer.

### Transferred R-iEOs promote enhanced T-cell infiltration into tumors

**Fig. 3:**
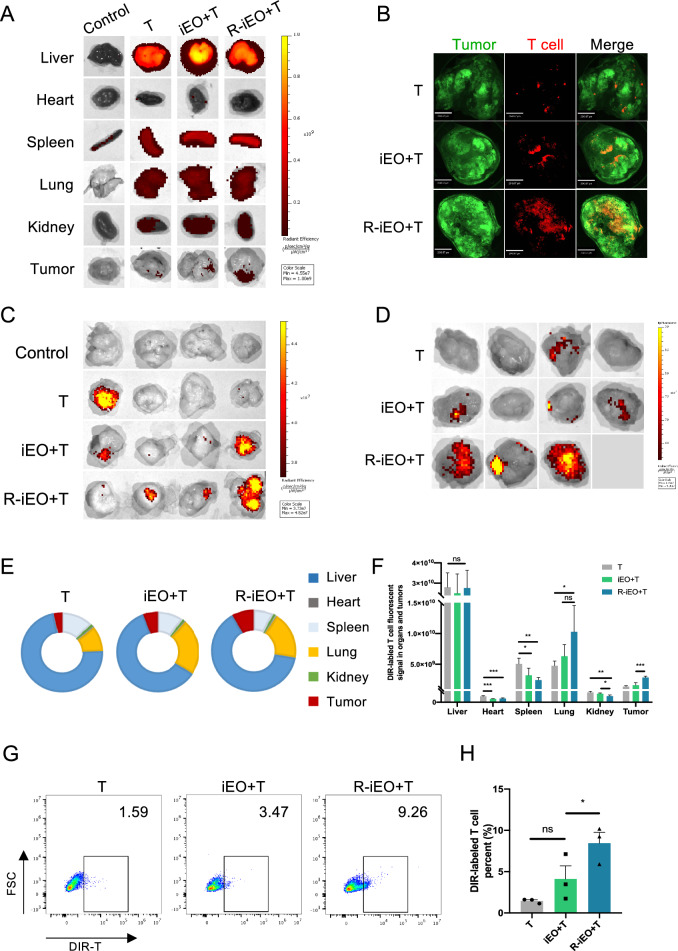
Enhanced T cell recruitment and infiltration into tumors with adoptive transferred R848-activated-eosinophils. **A** Representative images of different organs and tumors showing bio-distribution of DIR-labeled T cells at 24 h. **B** Representative confocal image of hyalinized tumors showing DIR-labeled T cells at 24 h. Scale bar represents 2043.67 μm. **C** Images of tumors showing accumulation of DIR-labeled T cells at 7d. **D** Accumulation of DIR-labeled T cells in large established tumors at 24 h. The gray image indicates the animal was dead before acquisition of image. **E** Tumor trafficking and biodistribution of DIR-labeled T cells in mice. (N = 3) **F** Quantitative analysis of fluorescence intensity for ex vivo imaging of organs and tumors in mice. (N = 3) **G**–**H** Flow cytometry analysis and quantification of T cells trafficking in organs in mice. The gating strategy is shown in Fig S2C. (N = 3) One-way ANOVA, *p* value * < 0.05, ** < 0.01, and *** < 0.001

Based on the increased infiltration of R-iEOs into the tumor site, we next sought to examine whether R-iEOs in tumors could further increase T-cell recruitment in vivo. We generated a tumor-bearing mouse model with HCT116 tumors (approximately 75 mm^3^ in size). Twenty-four hours after intravenous injection of R-iEOs or normal iEOs into the tumor-bearing mice, DiR-labeled CD8^+^ T cells were administered intravenously. After 24 h, the organs were collected and used to quantitatively analyze the distribution of DiR^+^ T cells. Very few fluorescent signals were detected in the tumor site after injections of T-cells alone, while eosinophil injection induced a much greater T-cell signal. The combination of T cells and R-iEOs resulted in robust T-cell infiltration in tumor sites (Fig. 3A). Confocal microscopy of hyalinized tumors confirmed that the greatest accumulation of T cells occurred in tumors after injection with a combination of T cells and R-iEOs (Fig. 3B).

To evaluate the persistence of the T-cell infiltration, we collected and imaged tumors from each group 7 days after CD8^+^ T-cell injection and found that all the samples showed low fluorescence signals. However, R-iEOs combined with T cells resulted in stronger T-cell infiltration than that in the normal iEOs or T cells groups (Fig. 3C).

We next developed a tumor-bearing mouse model with larger HCT116 tumors (approximately 1000 mm^3^ in size). Twenty-four hours after intravenous injection of R-iEOs or normal iEOs into mice, DiR-labeled CD8^+^ T cells were administered intravenously. DiR^+^ T cells were observed at tumor sites. The combination of R-iEOs with T cells increased the recruitment of T cells to tumors (Fig. 3D, E) but decreased T-cell accumulation in the spleen and kidney (Fig. 3F). Flow cytometry analysis of the lysed tumors further revealed a greater signal in the tumors of the R-iEO and T-cell combination group (Fig. 3G, H). The above data indicated that R-iEOs at tumor sites could enhance T-cell recruitment into larger well-established tumors.

### R-iEOs combined with CAR-T cells displayed enhanced antitumor activity

**Fig. 4:**
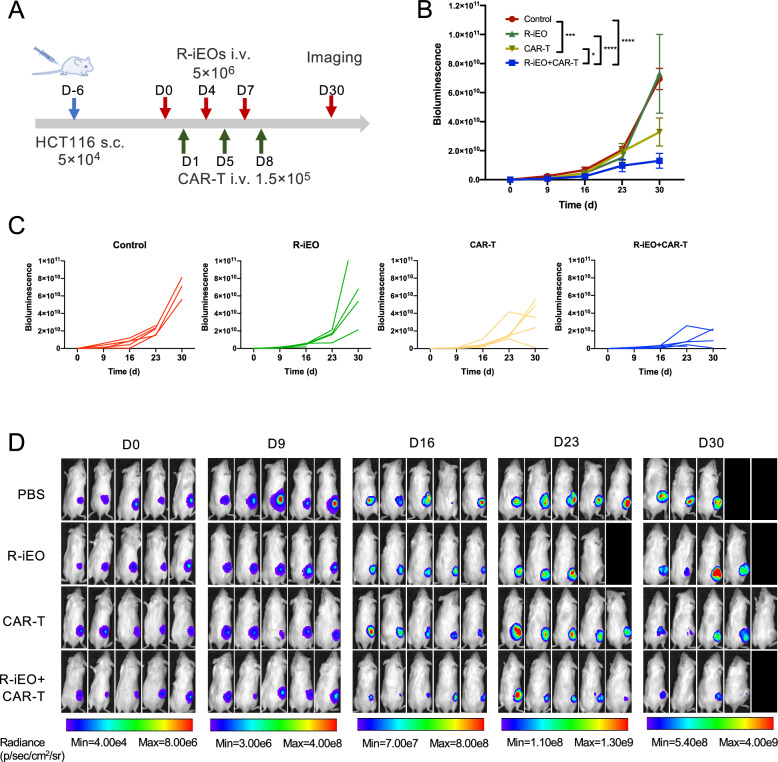
In vivo antitumor efficacy of functional R-iEOs combined with CAR-T cells. **A** In vivo antitumor efficacy study with a schematic diagram on the workflow. s.c., subcutaneous injection; i.v., intravenous injection. **B** Antitumor activity of R-iEOs combined with HER2-CAR-T cells in tumor-bearing mice by weekly in vivo bioluminescent imaging. Deceased animals are excluded from calculation in the following time points. One-way ANOVA was used for comparing bioluminescence measurements of each group at Day 30, *p* value * < 0.05, ** < 0.01, *** < 0.001, and **** < 0.0001. **C** in vivo bioluminescence curves of individual animals. Termination of curves represented the death of corresponding animals. **D** Photos of animals from different treatment groups. Black images are used after the death of the corresponding animals

We tested the effect of the combination of R-iEOs and CAR-T cells in vivo (Fig. 4A). HCT116 tumor cells were injected subcutaneously at a dose of 5 × 10^4^ per mouse. Three intravenous injections of R-iEOs were injected on day 0, day 4 and day 7 at a dose of 5 × 10^6^ to each mouse per injection. The transduction efficiency of CAR-T cells was assessed via flow cytometry analysis, and EGFP^+^ CAR-T cells were sorted via FACS before injection. The growth of HCT116 tumors treated with a combination of R-iEOs and CAR-T cells was significantly inhibited compared to that of tumors treated with CAR-T cells alone, although there was no significant difference between the single R-iEO treatment group and the control group (Fig. 4B–D).

### R848 activation led to type 1 polarization of iEOs

Although eosinophils have been studied mostly in the context of type 2 inflammatory responses such as allergies, they also participate in type 1 inflammatory responses [28]. Eosinophils display functional heterogeneity in response to different environmental triggers and release different cytokines [29]. Th1 cytokines such as TNF-α, can induce eosinophils to polarize to a proinflammatory type 1 phenotype. In contrast, exposure to Th2-associated cytokines (e.g., IL4 or IL13) drove eosinophils polarization toward type 2 eosinophils, resulting in an increase in IL4/13 signaling and genes associated with asthma [30].

**Fig. 5:**
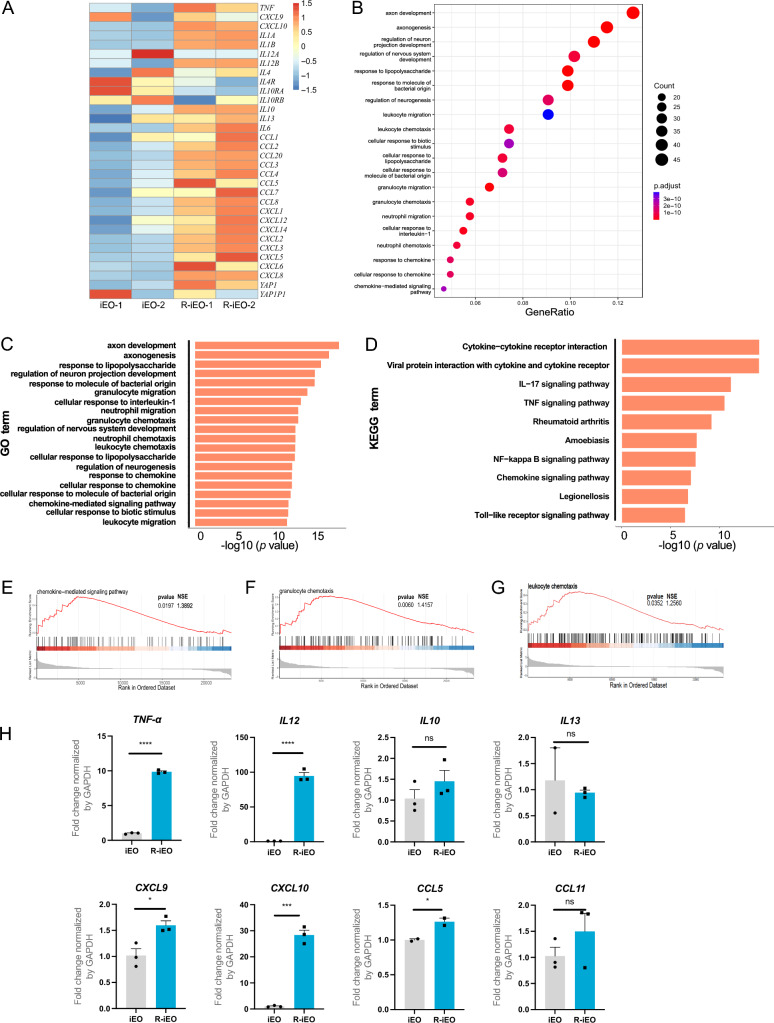
R-iEOs upregulate proinflammatory cytokines and chemokine signaling. **A** Heat map shows a distinct transcriptional state characterized by the up-regulation of genes involved in proinflammatory cytokine and chemokine signaling pathways. **B**, **C** Significantly enriched GO annotated classification distribution point map (**B**) and bar chart (**C**) are shown. **D** Significantly enriched KEGG pathways bar chart. **E**–**G** Expression changes in R-iEOs versus normal iEOs were compared by GSEA. Chemokine mediated signaling (**E**), Granulocyte chemotaxis (**F**), and leukocyte chemotaxis (**G**) are shown. **H** Q-PCR analysis confirmed the mRNA expression of proinflammatory cytokines and chemokines. (N = 3) Student’s T test, *p* value * < 0.05, *** < 0.001, and **** < 0.0001

To investigate the underlying mechanism and to characterize the molecular features of R-iEOs, we analyzed eosinophils using bulk RNA-seq. As shown in Fig. 5A, compared with untreated eosinophils, R848 treated eosinophils exhibited a distinct transcriptional state characterized by the upregulated expression of genes involved in proinflammatory cytokine signaling pathways and chemokine-mediated pathways. KEGG pathway analysis showed that key signaling pathways upregulated in R-iEOs included NF-κB, TNF, and IL17, indicating a broad activation of immune response in eosinophils (Fig. 5D). To further elucidate downstream pathways activated by R848 in eosinophils. We used specific inhibitors on pathways related to TLR signaling before and during R848 treatment. Q-PCR analysis revealed that inhibitors on component of inhibitor of nuclear factor kappa B kinase complex (IKK1), AP-1/NF-κB, and nuclear receptor subfamily 2 group C member 2 (TAK1) could drastically reduce the effect of R848 on eosinophils (Fig S5D, E). These results suggest that NF-κB signaling is a major downstream pathway governing eosinophil response toward R848.

R-iEOs increased the expression of cytotoxic Th1 immune response-related cytokines, such as *TNF, IL6*, *IL12, IL1A* and *IL1B* (Fig. 5A). Moreover, the exposure of iEOs to R848 led to increased expression of a wide range of chemokines, including *CXCL10*, a key factor that recruits effector T cells (Fig. 5A–H). Other chemokines, such as *CCL4* and *CCL5*, also had upregulated expression, which might be responsible for the chemotaxis of myeloid and lymphoid cells (Fig. 5A). On the other hand, R848-stimulated eosinophils caused a decrease in the expression of Th2 cytokine receptors, such as *IL4R* and *IL10Ra* (Fig. 5A). Taken together, the RNA-seq data demonstrated that in the presence of R848, eosinophils acquired a different transcription profile, which differentiated them into type 1, other than type 2 eosinophils.

To phenotypically characterize eosinophils post-R848 activation, a flow cytometry panel of eosinophil markers was used. As shown in Fig S1A-D, the expression of CD69, which is considered as a marker of eosinophil activation [31], was significantly greater in R848-treated eosinophils than in untreated eosinophils (Fig S1E). We also found a significant decrease in CD101 expression (Fig S1F), although the function of CD101 in eosinophils remains unclear. Recent research has suggested that homeostatic CD101^−^ eosinophils ameliorate neutrophilic inflammation in acute lung injury, while allergic CD101^+^ eosinophils exacerbate this inflammation [32]. Thus, the downregulation of CD101 expression in eosinophils likely suppresses the adverse effects of eosinophils in terms of lung injury.

To determine the difference in degranulation activity between the two eosinophil populations, we performed transmission electron microscopy (TEM) on eosinophils. Interestingly, we observed a significant change in the morphology of granules in the vesicles of R-iEOs, from large, intact granules in untreated eosinophils to small, dense granules in R-iEOs (Fig S1G, H). Thus, R848 stimulation allowed eosinophils to undergo a granule morphological transition.

### R-iEOs upregulated the expression of chemokines that directed T-cell recruitment

**Fig. 6:**
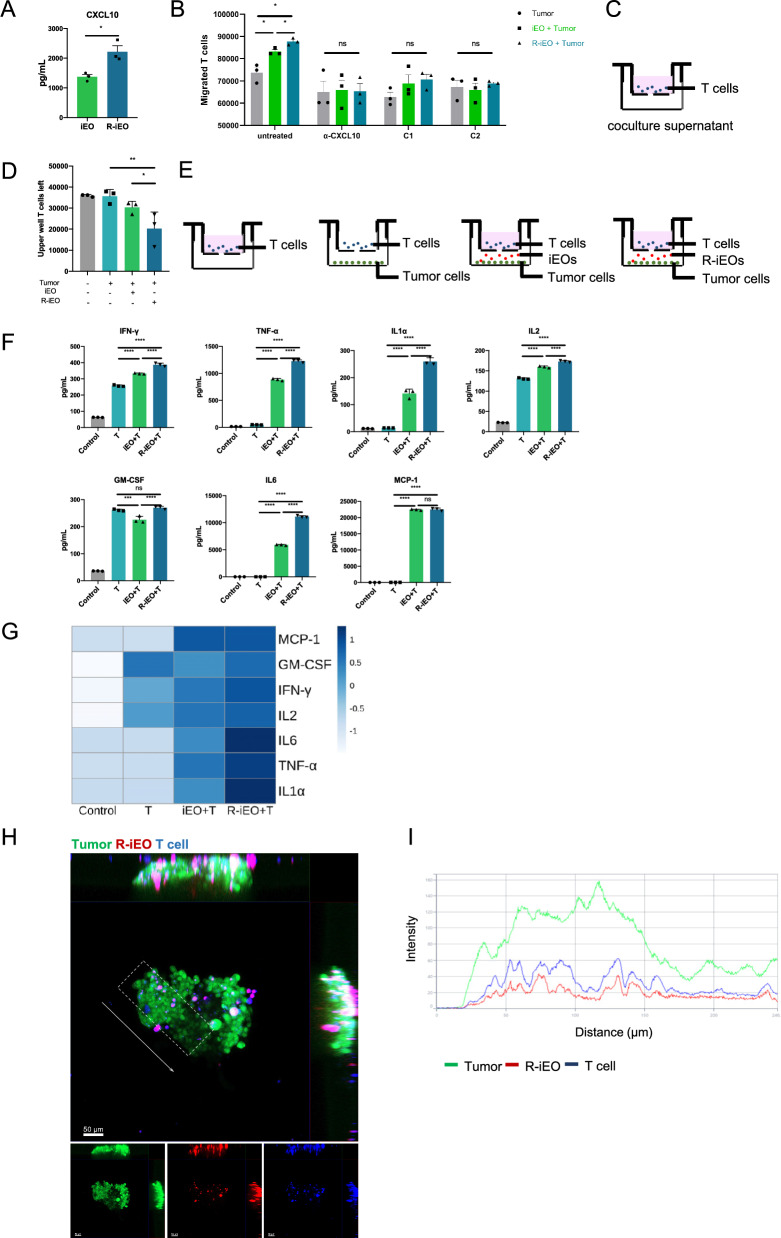
Upregulated proinflammatory cytokines and chemokine in R-iEOs combined with T cells. **A** ELISA analysis of CXCL10 secretion in the supernatant of R848-activated-eosinophils. (N = 3) Student’s T test, *p* value * < 0.05, **B**–**E** Quantification and schematic diagram of T cell migration model. Diagram of coculture supernatant of HCT116 tumor cells and iEOs or R-iEOs in the lower chamber (**C**) and iEOs or R-iEOs coculture with HCT116 tumor cells seeding in the lower chamber (**E**). T cells were added to the upper well of the transwell and were allowed to migrate for 6 h. (N = 3) One-way ANOVA, *p* value * < 0.05, ** < 0.01. **F**–**G** Luminex analysis (**F**) and heatmap (**G**) of cytokines and chemokines in the supernatant of T cells coculture with eosinophils and tumor cells. (N = 3) One-way ANOVA, *p* value * < 0.05, ** < 0.01, *** < 0.001, and **** < 0.0001. **H** Confocal microscopy showing colocalization of R-iEOs and T cells in vitro. Scale bar represents 50 μm. **I** Intensity profiling of R-iEOs (red) and T cells (blue) in tumor spheroid shows colocalized signal. The region of interest is presented as a white box in (**H**). The direction of the vector for distance is presented as a white arrow in **H**

In our previous study, we observed increased T-cells recruitment to the tumor site in vivo. To confirm the increase in T-cell recruitment in vitro, we used a transwell system for cell culture. We placed different supernatant media in the lower chamber and then added T cells to the upper chamber. As shown in Fig. 6A, CXCL10 release was significantly greater in the supernatant of R-iEOs than in that of normal iEOs. CXCL10 is thought to play an important role in directing T-cell recruitment. After 6 h of incubation, we counted the number of T cells that migrated through the membranes into the bottom chamber and found that coculture supernatant of HCT116 tumor cells and R-iEOs medium had a greater capacity to recruit the T cells than the coculture supernatant of tumor and normal iEO medium (Fig. 6B, C). Monoclonal antibodies against CXCL10 and inhibitors of CXCR3 significantly reduced T cell count of both eosinophil-tumor coculture group. Indicating CXCL10-CXCR3 axis as one of the key pathways in eosinophil promoted T cell tumor infiltration.

Furthermore, we placed HCT116 tumor cells with R-iEOs or normal iEOs in the lower chamber and then added CD8^+^ T cells to the upper chamber. After 6 h of incubation, the remaining CD8^+^ T cells in the upper chamber were counted (Fig. 6D, E). The remaining number of CD8^+^ T cells in the upper chamber was significantly lower in the R-iEO group than in the iEO group. These data confirmed that R-iEOs can recruit CD8^+^ T cells.

To assess cytokine release in the supernatant of R-iEOs cocultured with CD8^+^ T cells, a Luminex multiplex assay was performed. Compared with normal iEOs cocultured with CD8^+^ T cells, R-iEOs cocultured with CD8^+^ T cells showed increased levels of IFN-γ, TNF-α, IL1α, IL2, GM-CSF, and IL6, as compared to normal iEOs coculture with CD8^+^ T cells (Fig. 6F, G).

To investigate the effect of R-iEOs on the CD8^+^ T-cell infiltration in a more physiological context that better reflects the 3D architecture of the tumor, we performed live cell imaging assays using HCT116 tumor cell spheroids. GFP-transduced HCT116 tumor cells were added to low-attachment wells and left to form spheroids for 72 h, after which dye-labeled R-iEOs were added to the wells followed by the addition of dye-labeled CD8^+^ T cells added 1 h later. The colocalization of R-iEOs and CD8^+^ T cells was observed (Fig. 6H, I). Collectively, these data showed that R-iEOs increased the infiltration of CD8^+^ T cells into 3D tumor spheroids.

## Discussion

In this study, we developed a novel approach to harness the potential of the TLR7/8 agonist R848 to activate eosinophils derived from hCiPSCs. R-iEOs exhibited significantly reduced lung influx under inflammatory conditions, thereby alleviating safety concerns about the use of eosinophils for future potential applications. Ingeniously, R-iEOs not only preferentially accumulated in tumors but also promoted the infiltration of T cells into tumor sites. Notably, this effect could also be observed in larger tumor masses. The increased infiltration of R-iEOs and recruited T cells in solid tumors resulted in enhanced tumor suppression when R-iEOs were combined with CAR-T cells.

Our approach of generating improved eosinophils modulated with TLR7/8 signaling alleviated the safety concerns associated with eosinophils, advancing their potential application in combating solid tumors. In this study, by treating hCiPSC-derived iEOs with the TLR7/8 agonist R848, we found that these R-iEOs showed significantly reduced influx into the lungs under inflammatory conditions compared with untreated iEOs. Only 3% of R-iEOs had migrated into the lungs, while approximately 17% of iEOs had migrated into the lungs. R848 can prevent the influx of eosinophils into bronchoalveolar lavage fluid in a model of chronic asthma and can prevent the development of pulmonary pathology, such as airway hyperresponsiveness [24, 25, 33, 34]. Therefore, our study supports that modulating TLR7/8 is a promising target to control the influx of eosinophils into lungs.

Once the safety concerns of iEOs are mitigated, eosinophils will emerge as intriguing candidates alongside other innate myeloid cells for developing immunotherapies against solid tumors. Macrophages and neutrophils have also been generated from hiPSCs and the possibility of developing novel immunotherapies against solid tumors is being explored [35, 36]. These different innate myeloid cells have their respective advantages in regulating the immunity of solid tumor microenvironment [37–40]. More studies are needed to define the effects and underlying mechanisms of how these innate myeloid cells facilitate CAR-T therapy against solid tumors. Based on the improved safety profile of R-iEOs, it would be feasible to compare the efficacy of these innate myeloid cells for facilitating CAR-T immunotherapy in the future.

Not only was the safety concern alleviated, R-iEOs also exhibited enhanced infiltration into solid tumor sites and further exhibited enhanced efficacy in terms of recruiting more T cells. Currently, the development of an immunotherapeutic strategy involving CAR-T cells for solid tumors is still challenging due to the poor infiltration of CAR-T cells into solid tumors [2]. Eosinophils have been demonstrated to possess the unique advantages of quickly infiltrating tumors and further recruiting T cells [5]. However, the levels of infiltrated eosinophils and trafficked T cells are still low. In this study, after treating hCiPSC-derived eosinophils with R848, R-iEOs exhibited enhanced infiltration capacity into tumor sites by two folds (Fig. 2A–E) and attracted two times more T cells to tumors (Fig. 3A–C). This enhanced infiltration of T cells was also observed in larger well-established tumors (Fig. 3D). Consistently, more effective inhibition of solid tumors was observed in R-iEOs and CAR-T cell combination group (Fig. 4B). Therefore, our hCiPSC-derived iEOs further exhibited enhanced infiltration capacity and T-cell recruitment ability with R848 treatment, which made R-iEOs more attractive for further study against solid tumors.

Our study provides a new perspective to study the role of TLR7/8 signaling through the direct modulation of eosinophils to gain enhanced potency. The TLR7/8 agonist R848, known as resiquimod, is a member of the imidazoquinoline family and is an anti-inflammatory therapeutic agent that can be used to alleviate allergic asthma and chronic asthma via direct injection in mouse models. Following injection, R848 targets both Th1 and Th2 cytokine proteins in the lungs [34]. However, injection of R848 in mice can result in some side effects [41]. In this study, pretreatment of iEOs with R848 led to qualitative changes, resulting in a greatly reduced influx of R-iEOs into the lungs and enhanced infiltration of T cells into tumors.

RNA-seq analysis identified that upregulated chemokines could be the potential main factors leading to enhanced potency. Through confirmation by ELISAs and transwell assays, we observed that R-iEOs secreted 34% more CXCL10 than did iEOs, and blocking the CXCL10-CXCR3 axis deprived eosinophils of the promoting effect on T cell tumor trafficking. CXCL10, which binds to CXCR3 on activated T cells and promotes their directional trafficking [42, 43], is a major factor driving the attraction of T cells to tumor sites [44–46]. Additionally, CXCL10 also acts as a natural antagonist of CCR3 [47], even low dosages of it can block approximately 90% of pulmonary eosinophilia induced by eotaxin-2 [47, 48]. Thus, the increased expression of CXCL10 in R-iEOs may contribute to both the observed decrease in lung influx and the enhanced recruitment of T cells.

Promoting T cell infiltration into solid tumors is a crucial question for CAR-T immune therapy for solid tumors and has been widely studied. Chemokine signal is recognized as the crucial signal to promote T cell tumor infiltration into solid tumors [49], it might not be the sole leverage of eosinophil. Moreover, eosinophils could also modulate tumor microenvironment through cytokines and VEGF secretion [5]. These properties made eosinophils valuable for future studies on the potential efficacy to facilitate CAR-T cell infiltration and elimination of solid tumors.

R848 treatment induced major changes by the upregulation of genes involved in proinflammatory cytokine signaling pathways. For example, the concentration of IL6, TNF-α, and IL1α, etc. is increased in the supernatant of T cells upon coculture with R-iEOs (Fig. 6F). While these proinflammatory cytokines may enhance the effectiveness of CAR-T therapy, they may also trigger the cytokine release syndrome (CRS). In future clinical applications of R-iEOs, the intervention and therapy regarding the CRS could refer to CAR-T cell adoptive therapy by using anti-IL6 antibody (e.g., Tocilizumab), corticosteroids (e.g., dexamethasone) or other related approaches [50].

In this study, we aim to define safety risks related with eosinophils through a mouse model of allergic asthma because eosinophil-related asthma stood out to be the most concerned disease in eosinophil application. However, some questions still remain to be answered. First, as our study utilizes a murine model, the accumulation of human eosinophils may rely on the cross-reactivity of murine bio-molecules to human receptors [51]. More evidence with human patients is needed to provide an extensive safety profile. Second, the infiltration of eosinophils is not confined to the lung (Fig. 2). Other organs such as the skin, liver, and heart also suffer from eosinophil-mediated damages [52]. Third, while our study focused on the number of eosinophils in the allergenic airway as a key parameter related to asthma or damage level, we did not measure pathological changes of the airway in our experiments. A systematic study utilizing several allergic models of major organs focusing on pathological changes will be conducted in the future to show a more extensive safety profile for R-iEOs.

## Conclusions

Overall, our study on R848-activated eosinophils offers a strategy to mitigate the risk associated with the transfer of eosinophils. Additionally, our study suggests a potential solution to enhance T-cell infiltration into solid tumors. This approach of manipulating the iEO with R848 allowed us to “*hit two birds with one stone*”, addressing both aspects to improve the safety and the effectiveness of iEOs. Therefore, our study presents a promising strategy to enhance the efficacy of CAR-T-cell immunotherapy by combining CAR-T-cell therapy with innate immune cells for the treatment of solid tumors.

## Supplementary Information

Additional file 1.

Additional file 2.

Additional file 3.

Additional file 4.

Additional file 5.

Additional file 6.

Additional file 7.

Additional file 8.

## Data Availability

No datasets were generated or analysed during the current study.

## Abbreviations

hCiPSCs: Human chemically reprogrammed induced pluripotent stem cells
TLR: Toll-like receptor
iEOs: Induced eosinophils
R-iEOs: R848-activated iEOs
B-NHL: B-cell non-Hodgkin lymphoma
ICB: Immune checkpoint blockade
TEM: Transmission electron microscopy
CAR: Chimeric antigen receptor
ELISA: Enzyme-linked immunosorbent assay
GO: Gene ontology
KEGG: Kyoto encyclopedia of genes and genomes
GSEA: Gene set enrichment analysis
CRS: Cytokine release syndrome
MFI: Mean fluorescent intensity
