# Supplementary figures and images for: TLR7/8 signaling activation enhances the potency of human pluripotent stem cell-derived eosinophils in cancer immunotherapy for solid tumors
Source: Exp Hematol Oncol. 2025 Mar 1;14:26. doi: 10.1186/s40164-025-00613-y (PMC11871822; doi:10.1186/s40164-025-00613-y)

Supplementary Figure 1

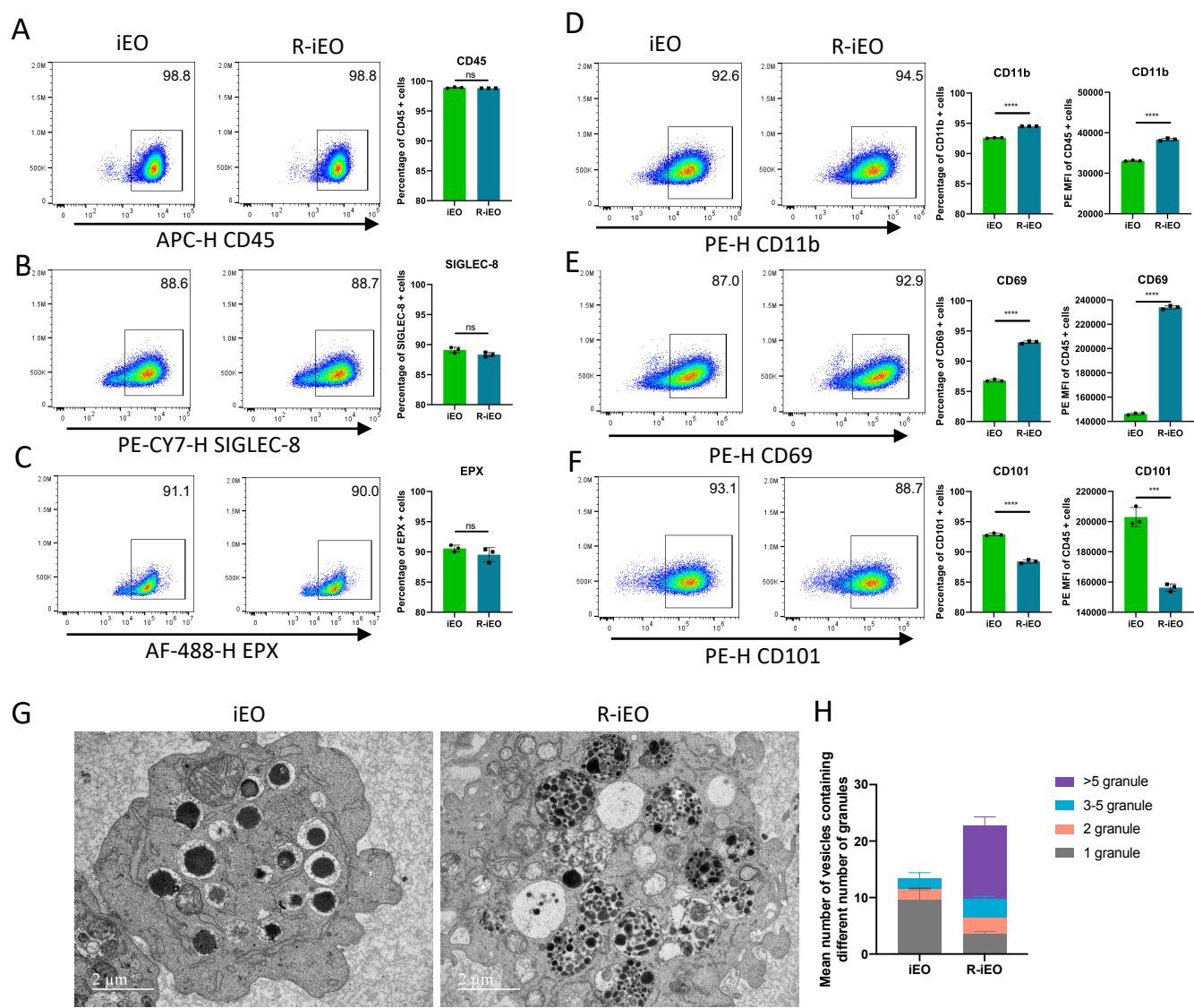

Supplement: Supplementary file 1 — Additional file 1. [file 40164_2025_613_MOESM1_ESM.pdf]

Supplementary Figure

A

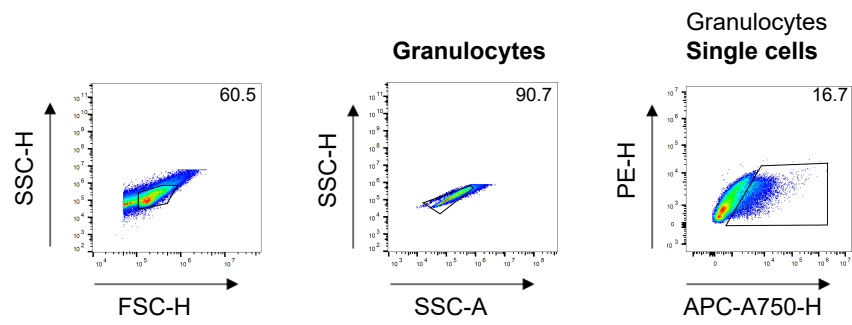

B

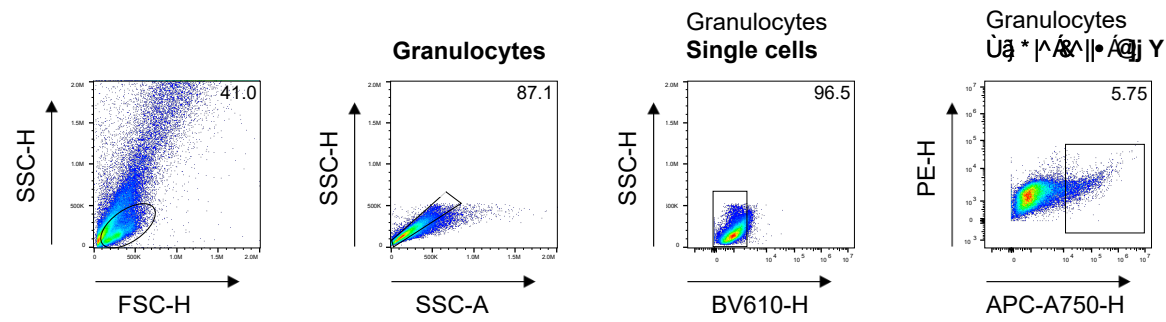

C

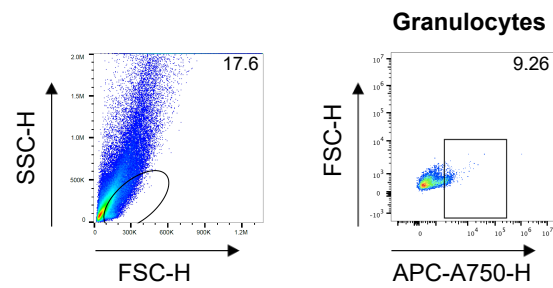

D

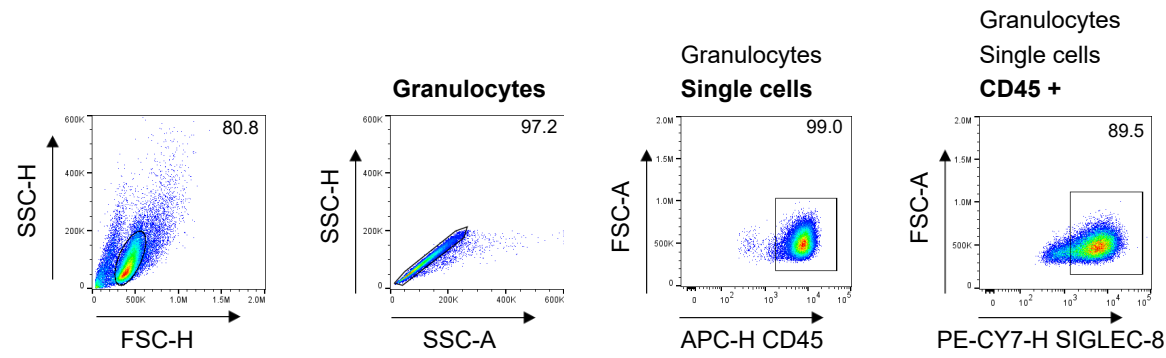

Supplement: Supplementary file 2 — Additional file 2. [file 40164_2025_613_MOESM2_ESM.pdf]

Supplementary Figure 3

A

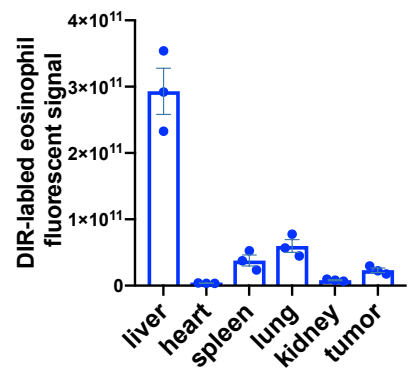

B

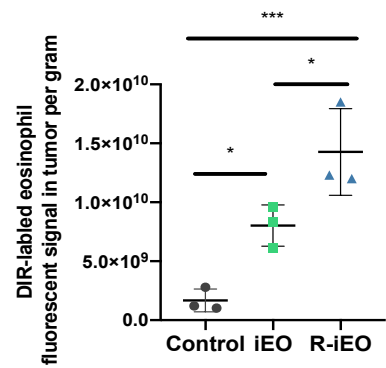

Supplement: Supplementary file 3 — Additional file 3. [file 40164_2025_613_MOESM3_ESM.pdf]

Supplementary Figure 4

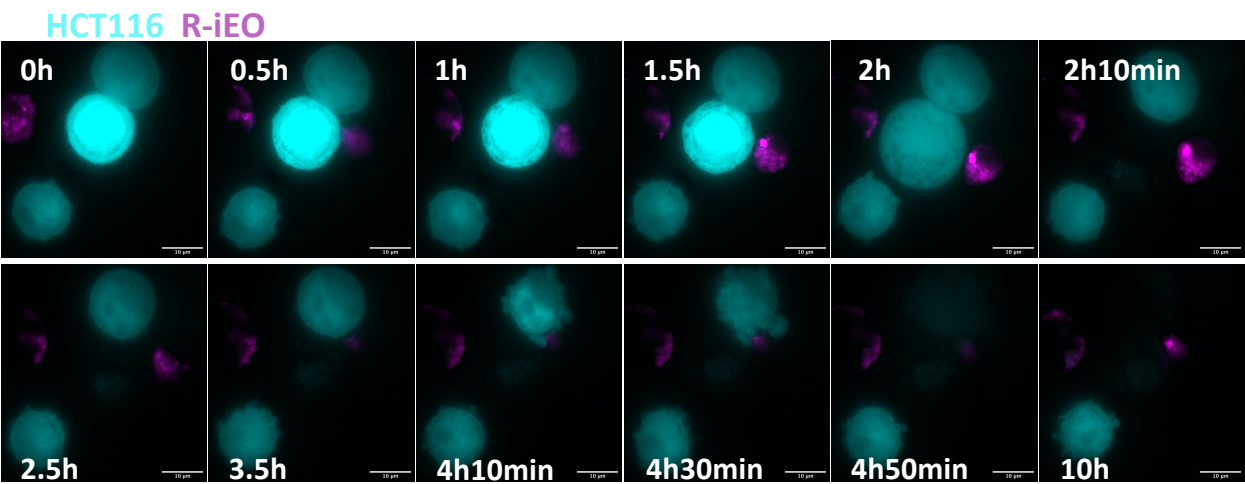

Supplement: Supplementary file 4 — Additional file 4. [file 40164_2025_613_MOESM4_ESM.pdf]

Supplementary Figure

A

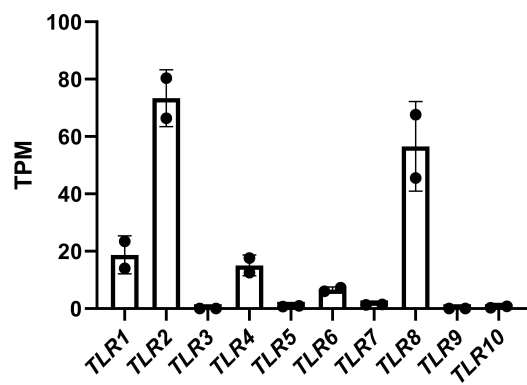

B

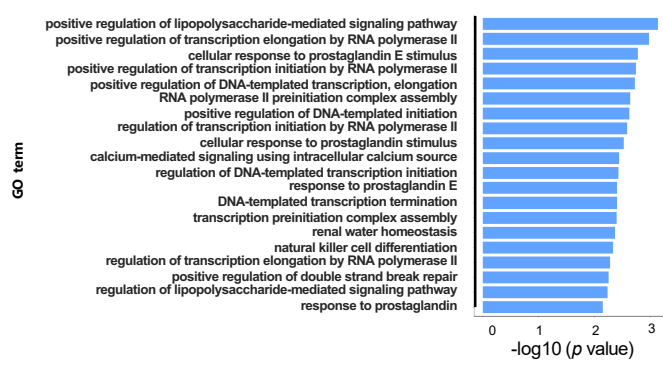

D

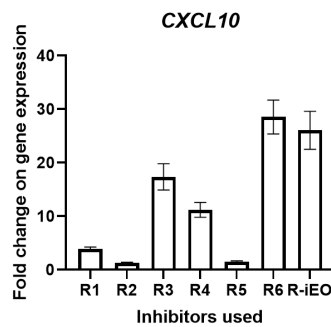

C

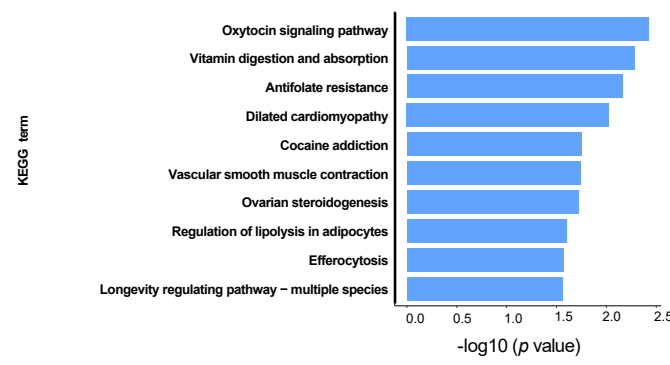

E

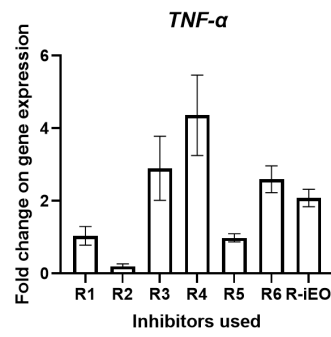

Supplement: Supplementary file 5 — Additional file 5. [file 40164_2025_613_MOESM5_ESM.pdf]

Supplementary Figure 6

A

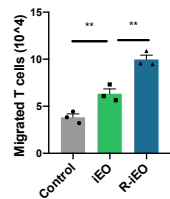

B

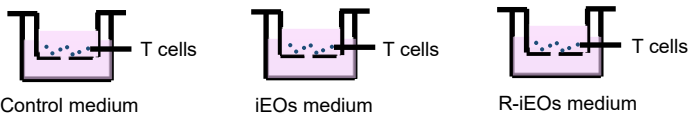

Supplement: Supplementary file 6 — Additional file 6. [file 40164_2025_613_MOESM6_ESM.pdf]
