# Supplementary material for: TLR7/8 signaling activation enhances the potency of human pluripotent stem cell-derived eosinophils in cancer immunotherapy for solid tumors
Source: Exp Hematol Oncol. 2025 Mar 1;14:26. doi: 10.1186/s40164-025-00613-y (PMC11871822; doi:10.1186/s40164-025-00613-y)
Supplement: Supplementary file 7 — Additional file 7. [file 40164_2025_613_MOESM7_ESM.pdf]

Supplementary Table 1. Primers for Q-PCR

CCL5- Forward	*ATGACTCCCGGCTGAACAAG*
CCL5- Reverse	*CTTTTGACAAAGCAGCGCCT*
CCL11- Forward	*GAAGTGGGTGCAGGATTCCA*
CCL11- Reverse	*GAACATTGCCCACACGTGAC*
CXCL9- Forward	*TGAGAAAGGGTCGCTGTTCC*
CXCL9- Reverse	*TCAAACTGCTTGGCTCACCA*
CXCL10- Forward	*GCTTCCAAGGATGGACCACA*
CXCL10- Reverse	*GCAGGGTCAGAACATCCACT*
TNF-α- Forward	*CTCGAACCCCGAGTGACAAG*
TNF-α- Reverse	*TGAGGTACAGGCCCTCTGAT*
IL-10- Forward	*TGCTCTTGCAAAACCAAACCA*
IL-10- Reverse	*GGGAGGTCAGGGAAAACAGC*
IL-13- Forward	*GGGATAAGGGGCGTTGACTC*
IL-13- Reverse	*CACAGTCTTCCCCAATCCCC*
IL-12- Forward	*CGCCTACTCTAGAGCTAGCGCCACC*
IL-12- Reverse	*GACTTCCTCTGCCCTCAGCGGCCGC*
