# Supplementary material for: TLR7/8 signaling activation enhances the potency of human pluripotent stem cell-derived eosinophils in cancer immunotherapy for solid tumors
Source: Exp Hematol Oncol. 2025 Mar 1;14:26. doi: 10.1186/s40164-025-00613-y (PMC11871822; doi:10.1186/s40164-025-00613-y)
Supplement: Supplementary file 8 — Additional file 8. [file 40164_2025_613_MOESM8_ESM.pdf]

Supplementary Table 2. Inhibitors used in Fig S5D, E and Fig 6B

| Inhibitor # | Name | CAS # | Cat. # | Working Conc. (μM) |
|---|---|---|---|---|
| R1 | BMS-345541 hydrochloride | 547757-23-3 | T8542 | 5 |
| R2 | AP-1/NF-κB activation inhibitor 1 | 188936-12-1 | T9656 | 1 |
| R3 | T-5224 | 530141-72-1 | T5416 | 20 |
| R4 | 666-15 | 1433286-70-4 | T5318 | 0.5 |
| R5 | Takinib | 1111556-37-6 | T4264 | 10 |
| R6 | IRAK-1-4 Inhibitor I | 509093-47-4 | T2457 | 10 |
| C1 | AMG 487 | 473719-41-4 | T10297L | 1 |
| C2 | rac-NBI-74330 | 473722-68-8 | T26035 | 1 |
